# Risk of glomerular filtration rate decline in patients with hypertrophic cardiomyopathy and obstructive sleep apnoea

**DOI:** 10.1038/s41598-017-17818-9

**Published:** 2017-12-12

**Authors:** Shao-Yun Wang, Jing Luo, Yi-Fei Dong, Xu-Yang Liu, Ying-Li Fan, Ming Deng, Da-Wei Chen, Ping Li, Xiao-Shu Cheng

**Affiliations:** 1 Department of Cardiovascular Medicine, the Second Affiliated Hospital of Nanchang University, Nanchang of Jiangxi, Jiangxi, China; 2 Key Laboratory of Molecular Biology in Jiangxi Province, Nanchang of Jiangxi, Jiangxi, China; 3 Department of Cardiovascular Medicine, the Affiliated Hospital of Jinggangshan University, Ji’an of Jiangxi, Jiangxi, China

## Abstract

Sleep apnoea is associated with chronic kidney diseases. A high obstructive sleep apnoea (OSA) prevalence is shown in patients with hypertrophic cardiomyopathy (HCM). Whether the presence of OSA would affect the renal function of patients with HCM is unknown. Forty-five consecutive patients with HCM were divided into the HCM OSA− and OSA+ groups. Forty-three patients with OSA without HCM were recruited as controls. Clinical indices, including estimated glomerular filtration rate (eGFR) and urine 8-hydroxy-2-deoxyguanosine (8-OHdG), were measured. The eGFR was significantly lower in the HCM OSA+ group than in the HCM OSA− (P < 0.05) and OSA (P < 0.001) groups. Multivariate linear regression analysis identified that the apnoea-hypopnoea index was independently associated with eGFR in all patients with HCM (β = −1.329, 95% confidence interval: −1.942, −0.717, P < 0.001). The urine 8-OHdG level, an oxidative stress marker, was significantly higher in the HCM OSA+ group than in the HCM OSA− (P < 0.001) and OSA (P < 0.001) groups and significantly correlated with the AHI (r = 0.467, P = 0.003) and eGFR (r = −0.457, P = 0.004) in all patients with HCM. Our study suggests a risk of eGFR decline in patients with HCM and OSA.

## Introduction

Hypertrophic cardiomyopathy (HCM) is the most common genetic cardiac disease and is an important cause of disability, including heart failure, atrial fibrillation, and sudden death in patients of all ages^1,2^. Novel evidence shows that obstructive sleep apnoea (OSA) is surprisingly common among patients with HCM, with a prevalence ranging from 32% to 71%, depending on the diagnostic criteria^3–7^. The presence of OSA among patients with HCM is independently associated with worse structural and functional impairments of the heart, including atrial and aortic enlargements^5^, worse New York Heart Association functional class^7^, and worse quality of life^8^. OSA is characterised by recurrent episodes of either partial or complete upper airway obstruction during sleep, leading to fragmented sleep and intermittent hypoxia^9^. Hypoxia and tubulointerstitial injury are common in all forms of kidney disease^10^. This has led to the “chronic hypoxia hypothesis”^11^, which emphasises ischemic damage in the tubulointerstitium as a final common pathway in end-stage kidney injury. In fact, accumulating evidence has shown a high prevalence of chronic kidney disease (CKD) in patients with OSA^12^. Conversely, previous studies further provided evidence that patients developed a high risk of OSA after kidney transplantation^13^, indicating a risk of OSA in patients with kidney diseases; moreover, percutaneous transluminal septal myocardial ablation could improve the renal function of patients with HCM^14^, indicating a risk of renal dysfunction in these patients. All of the abovementioned studies suggested a potential and important relationship among renal function, OSA, and HCM. Therefore, in the present study, we aimed to determine whether the presence of OSA would affect the renal function of patients with HCM and investigate the possible mechanism underlying this potential effect.

## Results

### Characteristics of the study groups

Table 1 details the characteristics of the study groups. The body mass index (BMI) was significantly lower in the HCM without OSA (HCM OSA−; P < 0.001) and HCM with OSA (HCM OSA+; P < 0.05) groups than in the OSA group. The HCM OSA+ group had a significantly higher BMI than the HCM OSA− group (P < 0.05). The 24-h mean diastolic blood pressure (DBP) was significantly higher in the OSA group (P < 0.05). The OSA and HCM OSA+ groups had significantly higher apnoea-hypopnoea index (AHI) and minimum O_2_ saturation than the HCM OSA− group. No difference in the AHI or minimum O_2_ saturation was found between the OSA and HCM OSA+ groups. The OSA group had a significantly higher rate of hypertension history (P < 0.001), while no difference was found between the HCM OSA− and HCM OSA+ groups. The HCM OSA− and HCM OSA+ groups took more beta-blockers (P < 0.001) and diuretics (P < 0.05) and fewer ACE-Is/ARBs (P < 0.05) and calcium-channel blockers (P < 0.05) than the OSA group. The HCM OSA− group took more anticoagulants than the OSA group (P < 0.05). The HCM OSA+ group had a significantly higher creatinine level than the HCM OSA− group (P < 0.05) and a significantly higher urea nitrogen level than the OSA group (P < 0.05). There were no significant differences in the 24-h mean heart rate, 24-h mean systolic blood pressure (SBP), atrial fibrillation (paroxysmal atrial fibrillation and persistent atrial fibrillation), reactive hyperemia index (RHI), high-sensitivity C-reactive protein (hs-CRP) level, and history of coronary heart disease, stroke, and diabetes among the three groups.

**Table 1:** Clinical characteristics of study groups.

Variable	All patients (n = 88)	OSA patients (n = 43)	HCM OSA− patients (n = 17)	HCM OSA+ patients (n = 28)
Gender, male, n (%)	61 (69.3%)	33 (76.7%)	8 (47.1%)	20 (71.4%)
Age, years	53.75 ± 13.33	50.79 ± 12.34	54.53 ± 15.17	57.82 ± 12.94
Body mass index, kg/m^2^	26.37 ± 5.92	29.20 ± 6.29	21.46 ± 3.62*	25.01 ± 3.71^†,§^
24 h mean heart rate, beats/min	73.01 ± 11.44	74.95 ± 10.44	70.41 ± 12.48	71.61 ± 12.15
24 h mean systolic blood pressure, mmHg	130.66 ± 22.75	137.33 ± 20.10	117.35 ± 18.62	128.50 ± 25.42
24 h mean diastolic blood pressure, mmHg	80.41 ± 15.32	86.51 ± 12.67	72.59 ± 13.43†	75.79 ± 16.72^†^
Atrial fibrillation, n (%)	18 (20.5%)	5 (11.6%)	6 (35.3%)	7 (25.0%)
Paroxysmal atrial fibrillation, n (%)	9 (10.2%)	3 (7.0%)	2 (11.8%)	4 (14.3%)
Persistent atrial fibrillation, n (%)	9 (10.2%)	2 (4.7%)	4 (23.5%)	3 (10.7%)
Current smoking, n (%)	23 (26.1%)	15 (34.9%)	3 (17.6%)	5 (17.9%)
Apnea-hypopnea index, events/h	24.56 ± 13.63	28.48 ± 13.78	7.69 ± 3.75*	28.77 ± 8.54^‡^
Minimum O_2_ saturation, %	81.41 ± 7.45	79.19 ± 7.44	87.41 ± 4.99^*^	81.18 ± 6.88^§^
Reactive hyperemia index	1.55 ± 0.53	1.66 ± 0.50	1.42 ± 0.41	1.46 ± 0.60
Medical history				
Hypertension, n (%)	54 (61.4%)	41 (95.3%)	3 (17.6%)^*^	10 (35.7%)*
Coronary heart disease, n (%)	3 (3.4%)	3 (7.0%)	0 (0%)	0 (0%)
Stroke, n (%)	9 (10.2%)	3 (7.0%)	2 (11.8%)	4 (14.3%)
Diabetes mellitus, n (%)	3 (3.4%)	0 (0%)	2 (11.8%)	1 (3.6%)
Medications				
Beta-blockers, n (%)	55 (62.5%)	13 (30.2%)	17 (100%)^*^	25 (89.3%)^*^
ACE-Is or ARBs, n (%)	49 (55.7%)	32 (74.4%)	5 (29.4%)^†^	12 (42.9%)^†^
Spironolactone, n (%)	21 (23.9%)	7 (16.3%)	4 (23.5%)	10 (35.7%)
Other diuretics, n (%)	22 (25.0%)	5 (11.6%)	6 (35.3%)^†^	11 (39.3%)^†^
Calcium-channel blockers, n (%)	41 (46.6%)	28 (65.1%)	3 (17.6%)^†^	10 (35.7%)^†^
Statins, n (%)	36 (40.9%)	20 (46.5%)	4 (23.5%)	12 (42.9%)
Anticoagulants, n (%)	13 (14.8%)	3 (7.0%)	6 (35.3%)^†^	4 (14.3%)
Blood biochemical indexes				
Fasting plasma glucose, mg/dL	4.88 (4.40,5.43)	5.12 (4.46,5.47)	4.63 (4.20,4.91)	4.83 (4.39,5.88)
Glycosylated hemoglobin, %	5.76 ± 0.79	5.83 ± 0.84	5.63 ± 0.75	5.74 ± 0.74
Total cholesterol, mmol/L	5.72 ± 0.83	5.83 ± 0.84	5.48 ± 0.77	5.69 ± 0.83
Triglyceride, mmol/L	2.28 ± 2.90	3.05 ± 3.94	1.51 ± 0.83	1.56 ± 0.74
HDL-C, mmol/L	1.08 ± 0.25	1.05 ± 0.28	1.05 ± 0.26	1.12 ± 0.18
LDL-C, mmol/L	2.38 ± 0.73	2.31 ± 0.66	2.24 ± 0.73	2.58 ± 0.81
Hs-CRP, mg/dL	2.77 (1.18,4.99)	2.97 (1.46,5.11)	1.27 (0.45,4.75)	2.84 (1.19,4.94)
Creatinine, µmol/L	74.10 (62.13,95.18)	70.50 (60.70,89.10)	66.60 (59.65,89.80)	88.55 (70.38,122.48)^§^
Urea nitrogen, mmol/L	5.99 ± 2.14	5.30 ± 1.63	5.77 ± 1.23	7.18 ± 2.73^†^
Uric acid, µmol/L	412.24 ± 111.65	401.98 ± 107.35	404.87 ± 143.95	432.48 ± 96.61
Estimated GFR, ml/min per 1.73 m^2^	112.22 ± 33.47	123.21 ± 30.82	116.82 ± 25.34	92.55 ± 33.84^*§^

Abbreviations: ACE-Is, angiotensin-converting enzyme inhibitors; ARBs, angiotensin II receptor blockers; GFR, glomerular filtration rate; Hs-CRP, high-sensitivity c-reactive protein; HDL-C, high-density lipoprotein cholesterol; HCM, hypertrophic cardiomyopathy; LDL-C, low-density lipoprotein cholesterol; OSA, obstructive sleep apnea.

*P < 0.001, ^†^ *P* < 0.05 compared patients with OSA; ^‡^ *P* < 0.001, ^§^ *P* < 0.05 compared patients with HCM.

### Echocardiography

Table 2 details the echocardiographic data of the three groups. The HCM OSA− and HCM OSA+ groups had significantly larger left atrial diameter (P < 0.05) and higher interventricular septal wall end-diastolic thickness (SWTd) (P < 0.001), left ventricular mass index (LVMI) (P < 0.001), and percentage of left ventricular outflow tract obstruction (P < 0.001) than the OSA group. No significant differences in the echocardiographic data were found between the HCM OSA− and HCM OSA+ groups.

**Table 2:** Echocardiographic data.

Variable	All patients (n = 88)	OSA patients (n = 43)	HCM OSA− patients (n = 17)	HCM OSA+ patients (n = 28)
Left atrial diameter, mm	37.70 ± 6.44	34.79 ± 4.97	41.53 ± 6.60†	39.86 ± 6.47^†^
Right atrial diameter, mm	36.00 (34.00,39.00)	35.00 (32.00,38.00)	37.00 (35.00,40.00)	37.00 (36.00,39.00)
Left ventricular end-diastolic diameter, mm	46.24 ± 7.71	47.19 ± 7.29	43.76 ± 7.54	46.29 ± 8.37
Right ventricular end-diastolic diameter, mm	22.00 (20.00,23.00)	22.00 (20.00,23.00)	22.00 (17.50,24.50)	22.00 (20.25,23.00)
Interventricular septal wall end-diastolic thickness, mm	14.66 ± 4.70	11.05 ± 1.85	19.59 ± 2.87*	17.21 ± 4.24*
Left ventricular end-diastolic posterior wall thickness, mm	10.00 (9.00,12.00)	10.00 (9.00,11.00)	11.00 (9.00,13.50)	10.50 (10.00,12.00)
Ascending aorta diameter, mm	31.85 ± 4.30	32.21 ± 4.18	29.65 ± 4.21	32.64 ± 4.25
Left ventricular mass index, g/m^2^	110.44 (86.41,147.27)	87.36 (75.14,104.11)	179.73 (126.63,215.23)*	132.55 (117.45,163.28)*
Left ventricular ejection fraction, %	62.00 (58.00,69.00)	61.00 (58.00,67.00)	60.00 (58.00,69.00)	67.00 (58.00,69.00)
Left ventricular outflow tract obstruction, %	21 (23.9%)	0 (0%)	8 (47.1%)*	13 (46.4%)*

Abbreviations: HCM, hypertrophic cardiomyopathy; OSA, obstructive sleep apnea.

*P < 0.001, ^†^ *P* < 0.05 compared subjects with OSA.

### Significantly lower eGFR in the HCM OSA+ group

The HCM OSA+ group showed a significantly lower eGFR level than the HCM OSA− (P < 0.05) and OSA (P < 0.001) groups (Table 1).

### AHI independently correlated with eGFR

Univariate and stepwise multivariate linear regression analyses were performed in the patients with HCM with and without OSA (Table 3). The univariate linear regression analysis showed that the heart rate (β = −1.469, 95% confidence interval [CI]: −2.167, −0.772, P < 0.001), AHI (β = −1.677, 95% CI: −2.297, −1.057, P < 0.001), use of calcium-channel blockers (β = −25.263, 95% CI: −45.873, −4.652, P = 0.017), urea nitrogen level (β = −6.707, 95% CI: −10.445, −2.970, P = 0.001), and history of hypertension (β = −22.208, 95% CI: −43.148, −1.267, P = 0.038) were significantly correlated with the eGFR. The multivariate linear regression analysis further identified that the AHI (β = −1.329, 95% CI: −1.942, −0.717, P < 0.001) and heart rate (β = −0.956, 95% CI: −1.587, −0.325, P = 0.004) were independently and significantly correlated with the eGFR (model R^2^ = 0.517).

**Table 3:** Univariate and stepwise multivariate of linear regression analyses for eGFR in HCM patients with or without OSA.

variable	univariate analysis		multivariate analysis	
	β(95% CI)	P value	β(95% CI)	P value
Gender, male	−7.247 (−27.718, 13.223)	NS (0.479)	not selected	
Age	−0.224 (−0.955, 0.507)	NS (0.539)	not selected	
Body mass index	−0.073 (−2.576, 2.431)	NS (0.954)	not selected	
Heart rate	−1.469 (−2.167, −0.772)	<0.001	−0.956 (−1.587, −0.325)	0.004
Reactive hyperemia index	−6.862 (−25.264, 11.918)	NS (0.465)	not selected	
Current smoking	−4.487 (−30.563, 21.589)	NS (0.730)	not selected	
Apnea-hypopnea index	−1.677 (−2.297, −1.057)	<0.001	−1.329 (−1.942, −0.717)	<0.001
Minimum O_2_ saturation	1.227 (−0.190, 2.644)	NS (0.088)	not selected	
Diabetes mellitus	19.399 (−20.239, 58.917)	NS (0.330)	not selected	
Hypertension	−22.208 (−43.148, −1.267)	0.038	—	
Atrial fibrillation	−6.513 (−28.448, 15.422)	NS (0.552)	not selected	
Left ventricular outflow tract obstruction	11.396 (−8.306, 31.098)	NS (0.250)	not selected	
Statins	−16.673 (−36.888, 3.543)	NS (0.104)	not selected	
ACE-Is or ARBs	−4.588 (−25.131, 15.954)	NS (0.655)	not selected	
Spironolactone	−7.696 (−29.130,13.739)	NS (0.473)	not selected	
Other diuretics	−2.991 (−23.562, 17.579)	NS (0.771)	not selected	
Calcium-channel blockers	−25.263 (−45.873, −4.652)	0.017	—	
Beta-blockers	28.172 (−10.901, 67.245)	NS (0.153)	not selected	
Anticoagulant	−0.751 (−24.763, 23.262)	NS (0.950)	not selected	
Ln Fasting plasma glucose	7.622 (−40.897, 56.141)	NS (0.753)	not selected	
Ln Glycosylated hemoglobin	42.593 (−34.643, 119.830)	NS (0.272)	not selected	
Triglyceride	2.502 (−10.722, 15.726)	NS (0.705)	not selected	
Total cholesterol	5.400 (−6.974, 17.744)	NS (0.384)	not selected	
HDL-C	−4.412 (−51.845, 43.021)	NS (0.852)	not selected	
LDL-C	−1.956 (−14.749, 10.836)	NS (0.759)	not selected	
Ln Hs-CRP	−5.461 (−16.625, 5.704)	NS (0.329)	not selected	
Urea nitrogen	−6.707 (−10.445, −2.970)	0.001	—	
Uric acid	−0.063 (−0.148, 0.022)	NS (0.143)	not selected	
Ln LVEF	−4.371 (−54.055, 45.313)	NS (0.860)	not selected	
Ln LVMI	−12.598 (−38.223, 13.027)	NS (0.327)	not selected	

Dashes indicate that the variable did not enter multivariate stepwise linear regression model. Abbreviations: ACE-Is, angiotensin-converting enzyme inhibitors; ARBs, angiotensin II receptor blockers; eGFR, estimated glomerular filtration rate; HDL-C, high-density lipoprotein cholesterol; Hs-CRP, high-sensitivity c-reactive protein; HCM, hypertrophic cardiomyopathy; LVEF, left ventricular ejection fraction; LVMI, left ventricular mass index; LDL-C, low-density lipoprotein cholesterol; OSA, obstructive sleep apnea.

### Significantly higher 8-OHdG levels in the HCM OSA+ group

**Figure 1:**
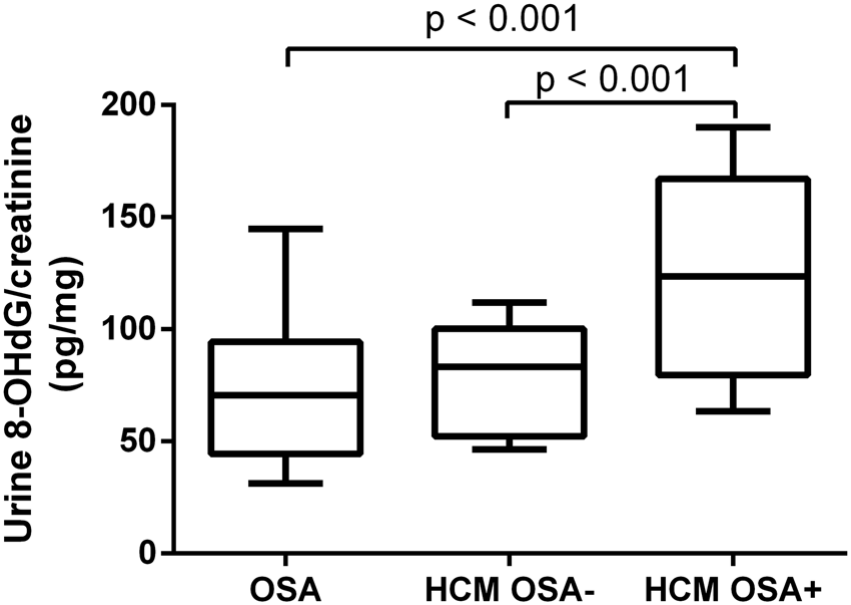
Creatinine-adjusted urine 8-OHdG levels in OSA, HCM OSA− and HCM OSA+ patients. 8-OHdG, 8-hydroxy-2-deoxyguanosine; HCM, hypertrophic cardiomyopathy; OSA, obstructive sleep apnea.

Figure 1 shows that the creatinine-adjusted urine 8-OHdG level was significantly higher in the HCM OSA+ group than in the HCM OSA− (P < 0.001) and OSA (P < 0.001) groups.

### 8-OHdG level correlated with the AHI and eGFR

**Figure 2:**
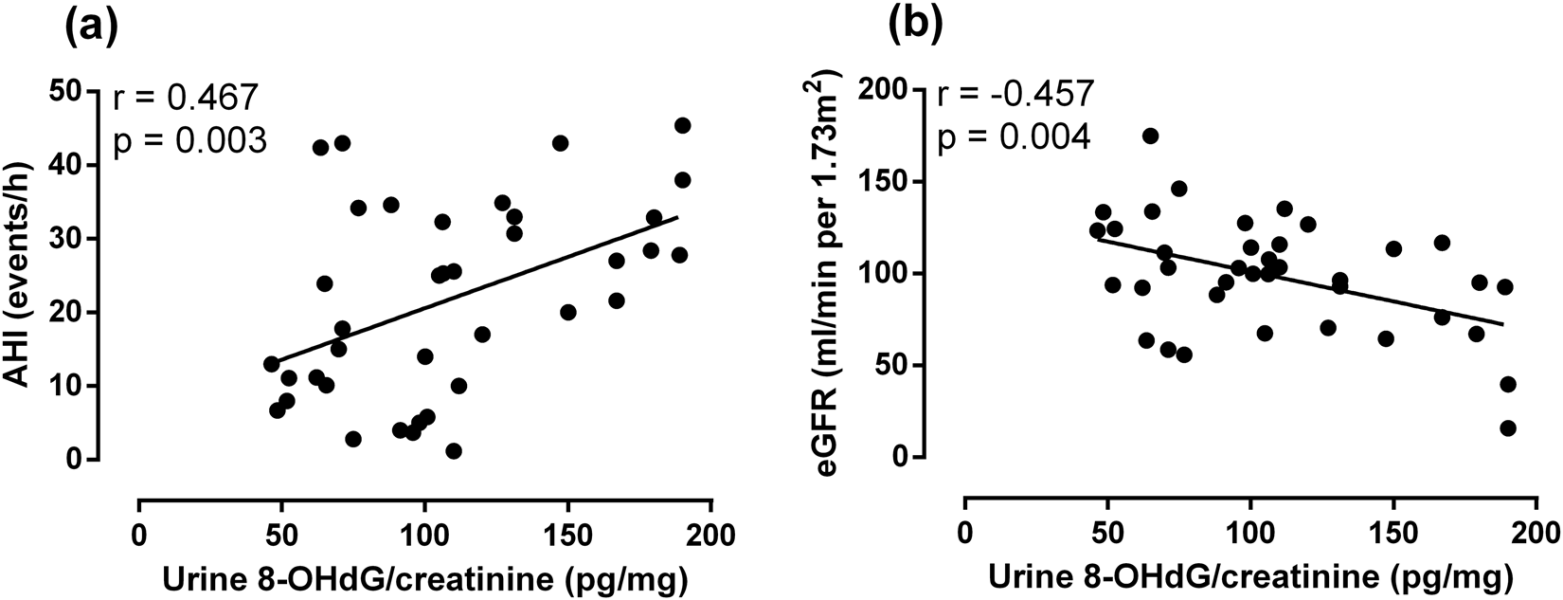
Correlation of creatinine-adjusted urine 8-OHdG level with the severity of AHI and eGFR. Figure 2a shows the correlation between creatinine-adjusted urine 8-OHdG level and the severity of AHI in all HCM patients. Figure 2b shows the correlation between creatinine-adjusted urine 8-OHdG level and eGFR in all HCM patients. 8-OHdG, 8-hydroxy-2-deoxyguanosine; AHI, apnea-hypopnea index; eGFR, estimated glomerular filtration rate; HCM, hypertrophic cardiomyopathy; OSA, obstructive sleep apnea.

Figure 2a shows that the creatinine-adjusted urine 8-OHdG level was positively correlated with the severity of the AHI in the patients with HCM with and without OSA (r = 0.467, P = 0.003). Figure 2b shows that the creatinine-adjusted urine 8-OHdG level was negatively correlated with the eGFR in the patients with HCM with and without OSA (r = −0.457, P = 0.004).

## Discussion

A major finding in the present study was that the presence of OSA was associated with an eGFR decline in the patients with HCM, which was accompanied by an increased urine 8-OHdG level. This finding was supported by the following results: 1) a significantly lower eGFR was shown in the HCM OSA+ group than in the OSA and HCM OSA− groups (Table 1); 2) the severity of the AHI was independently and negatively correlated with the eGFR in all patients with HCM (Table 3); 3) a significantly higher creatinine-adjusted urine 8-OHdG level was shown in the HCM OSA+ group than in the OSA and HCM OSA− groups (Fig. 1); and 4) the creatinine-adjusted urine 8-OHdG level was correlated significantly with the severity of the AHI and eGFR in all patients with HCM (Fig. 2).

The finding in the present study may help bridge the gap in understanding the effect of OSA on the eGFR of patients with HCM. Accumulating evidence has indicated a bidirectional interaction between OSA and renal function^15–17^. Conversely, there is an increased risk of OSA in patients with CKD;^16^ further, the rates of CKD are also greater in patients with OSA^15^, raising the possibility that sleep apnoea can contribute to CKD development as well. Considering the surprisingly high prevalence of OSA in patients with HCM^3–7^, we hypothesised that the eGFR would most probably be affected by the presence of OSA in these patients. In the present study, a significantly lower eGFR was shown in the HCM OSA+ group. Furthermore, the severity of the AHI was independently associated with the decrease in the eGFR in the patients with HCM with and without OSA. These results indicate that the presence of OSA, as a highly prevalent associated complication in HCM, might increase the risk of eGFR decline in this population.

The hypoxia and reoxygenation cycles in OSA cause a change in the oxidative balance, leading to an increasing formation of reactive oxygen species (ROS)^18^. Although there is no consensus concerning the pathogenesis of oxidative stress in OSA itself and its related organic disorders, measurement of the markers of oxidative stress in OSA may be a contributing aspect to the assessment and monitoring of patients, both with respect to the severity of the disease and the effectiveness of therapy^19^. We hypothesised that the coexistence of OSA in HCM would disrupt the balance between ROS removal and formation to initiate oxidative stress and constitute a pathogenesis for the decrease in eGFR in patients with HCM. ROS induce several types of DNA damage, such as strand breaks, base modifications, and cross-linking between DNA and various proteins^20^. By inducing hydroxylation of the C-8 position of 2′-deoxyguanosine, ROS produce 8-OHdG. This modified DNA base has recently been reported to be a reliable marker of oxidative DNA damage when measured in the tissues and urine^21^. The severity of OSA was previously reported to be independently correlated with the 8-OHdG level^22^ but not with other oxidative stress markers, such as thiobarbituric acid reactive substances, oxidised low-density lipoprotein cholesterol (LDL-C), and isoprostanes in humans^23^. Thus, we measured the urine 8-OHdG level in the patients to evaluate whether the urine 8-OHdG level would correlate with the severity of the AHI and eGFR and whether the coexistence of OSA would increase the urine 8-OHdG level in the patients with HCM. As shown in Fig. 1, the creatinine-adjusted urine 8-OHdG level significantly increased in the HCM OSA+ group compared with that in the HCM OSA− and OSA groups. Furthermore, the creatinine-adjusted urine 8-OHdG level was positively correlated with the AHI (Fig. 2a) and negatively correlated with the eGFR (Fig. 2b) in the patients with HCM with and without OSA, which suggests a relationship between oxidative stress and OSA and eGFR, respectively, in the patients with HCM. Importantly, these results indicate that oxidative stress might be one of the possible mechanisms underlying the effect of OSA on the eGFR of patients with HCM.

There were some other points worth discussing in the present study. Firstly, the prevalence of OSA in the patients with HCM was 62.2% in the present study. In previous studies, the prevalence had a vast range because the diagnostic criteria were different^3–7^. We defined OSA as an AHI of ≥15 events/h according to a previous study on patients with HCM^5^. Secondly, evidence indicates that patients with HCM and OSA are typically less obese than patients with OSA observed in sleep clinics^5,7^. Again, our results showed that the BMI of the HCM OSA+ group was significantly lower than that of the OSA group (Table 1). Thirdly, the interventricular SWTd and LVMI tended to be lower (Table 2) in the HCM OSA+ group, although there was no significant difference between the HCM OSA− and HCM OSA+ groups. The reason for such was unclear. However, the coexistence of OSA might urge patients to seek diagnosis and treatment earlier than patients with only HCM because of the symptoms associated with OSA itself. Finally, some findings in the present study were inconsistent with those of previous studies^6,24,25^. We found that the RHI, which indicates endothelial function, was not significantly different between the HCM OSA− and HCM OSA+ groups (Table 1). Further, we found that the rate of atrial fibrillation in the HCM OSA+ group was not significantly different from that in the HCM OSA− group. The endothelial function (represented as the RHI) in the present study was evaluated by non-invasively measuring the arterial tone changes in the peripheral arterial beds, which was different from the test using intra-arterial infusion of acetylcholine and forearm blood flow measurement in a previous study on patients with OSA^24^. Furthermore, the relatively lower left atrial and LVMI in the HCM OSA+ group than in the HCM OSA− group might affect the incidence of atrial fibrillation in the HCM OSA+ group in the present study.

The present study has certain limitations. Firstly, our study has a cross-sectional design; therefore, we could not investigate the cause and effect association between OSA and eGFR in the patients with HCM. Furthermore, we did not evaluate the effect of the association between OSA and eGFR on cardiovascular events in these patients. Thus, future prospective studies that would further investigate these issues are warranted. Secondly, the values of certain parameters were probably influenced by the medications to an extent. Thirdly, the subjects were limited to patients with OSA, and the role of central sleep apnoea in HCM still needs future investigations. In addition, the study investigated only a relatively small number of patients in a single centre. The mechanism underlying the effect of OSA on the eGFR of patients with HCM remains unclear.

Taken together, our study provided the first evidence for the effect and possible mechanism of OSA on the eGFR of patients with HCM despite the abovementioned limitations. The results indicate that the presence of OSA might increase the risk of eGFR decline in patients with HCM. Thus, future prospective studies are warranted to clarify this issue.

## Methods

### Patients

We evaluated 45 consecutive patients diagnosed with HCM in the Department of Cardiovascular Medicine at the Second Affiliated Hospital of Nanchang University from January 2015 to January 2016. All patients underwent polysomnography (PSG) and were divided into the HCM OSA+ and HCM OSA− groups based on their AHI (≥15 or <15 events/h)^5^. OSA was diagnosed in accordance with the Chinese Guidelines for the Diagnosis and Treatment of Obstructive Sleep Apnea Hypopnea Syndrome (Revised Edition 2011)^26^. Forty-three patients diagnosed with OSA without HCM matching the severity of AHI in the HCM OSA+ group (AHI ≥ 15 events/h) at the same period were recruited as controls. HCM was diagnosed in accordance with the 2014 ESC Guidelines on the diagnosis and management of HCM^27^. For patients with hypertension, HCM was diagnosed only when the patients met the following criteria: 1) family history of HCM; 2) maximum left ventricular wall thickness of ≥15 mm; and 3) marked repolarisation abnormalities, conduction disease, or Q-waves on 12-lead electrocardiogram^27^.

The exclusion criteria were as follows: history of continuous positive airway pressure therapy, central sleep apnoea, acute myocardial infarction, acute heart failure, severe respiratory insufficiency, severe liver disease, systemic or local inflammatory, cancer, and refusal to participate in the study.

The study was approved by the Medical Research Ethics Committee of the Second Affiliated Hospital of Nanchang University, and a signed informed consent was obtained from each patient before participation. All methods were performed in accordance with the relevant guidelines and regulations.

### Clinical indices

Doppler echocardiography was performed to evaluate the cardiac structure and function as described previously^28^. All echocardiographic examinations were performed using the Siemens-Acuson Sequoia^TM^ 512 ultrasound machine (Siemens, Erlangen, Germany) with a curved array multifrequency transducer (2.25–4.25 MHz) by experienced sonographers who were blinded to the patients’ clinical characteristics. Two-dimensional and two-dimensionally guided M-mode images were recorded from standardised views. Left atrial diameter, right atrial diameter, left ventricular end-diastolic diameter (LVIDd), left ventricular end-diastolic posterior wall thickness (PWTd), right ventricular end-diastolic diameter, interventricular SWTd, ascending aortic diameter, LVMI, left ventricular ejection fraction, and left ventricular outflow tract obstruction were measured. Left ventricular outflow tract obstruction was defined as an instantaneous peak Doppler left ventricular outflow tract pressure gradient of ≥30 mmHg at rest or during physiological provocation, such as Valsalva manoeuvre, standing, and exercise. Left ventricular mass was calculated using the formula: Left ventricular mass = 0.8 × {1.04[(LVIDd + PWTd + SWTd)^3^ − (LVIDd)^3^]} + 0.6 g^29^. LVMI was calculated by dividing the left ventricular mass by the body surface area. The body surface area was calculated as follows: 0.0073 × (height in centimetre) + 0.0127 × (weight in kilogram) − 0.2106 (for women) and 0.0057 × (height in centimetre) + 0.0121 × (weight in kilogram) + 0.0882 (for men)^30^.

All participants underwent overnight PSG (PHILIPS RESPIRONICS, Alice PDx, 1001 Murry Ridge Lane Murrysville, PA, USA). PSG was performed and scored in accordance with the American Academy of Sleep Medicine (AASM) practice standards. OSA was defined as a drop of ≤90% in the airflow at baseline for ≥10 s as recorded using an oronasal sensor with continued respiratory effort. Apnoea was defined as the complete cessation of airflow or a clear decrease in airflow of ≥90% lasting for ≥10 s. Hypopnoea was defined as a clear decrease in airflow of ≥50% lasting for ≥10 s accompanied by a decrease in blood oxygen saturation (SpO_2_) of at least 3% or a clear decrease in airflow of ≥30% lasting for ≥10 s accompanied by a decrease in SpO_2_ of at least 4% and/or associated with arousal. The average number of apnoeas and hypopnoeas per hour of sleep was defined as the AHI. Nocturnal oxygen desaturation was assessed as the minimum O_2_ saturation during sleep. The diagnosis and severity of OSA were based on the definitions recommended by the AASM as follows: non-OSA (AHI, <5 events/h), mild OSA (AHI, 5–15 events/h), moderate OSA (AHI, 15–30 events/h), and severe OSA (AHI, ≥30 events/h). Patients with AHI of ≥15 events/h were recruited in the present study.

eGFR was calculated on the basis of the modified glomerular filtration rate estimating equation for Chinese patients with CKD: eGFR MDRD = 186 × (serum creatinine in mg/dL)^−1.154^× (age in years)^−0.203^ × 0.742 (in women) × 1.233^31^.

Urine samples were successfully obtained from 39 patients with OSA, 14 patients with HCM OSA−, and 24 patients with HCM OSA+ . The urine 8-OHdG level was measured using a commercial ELISA kit (Cloud-Clone Corp, Suite 226, Houston, TX, USA) in Test Centre of Cloud-Clone Corp, WUHAN, China (Export Processing Zone, WUHAN, Hubei 430056, PRC). The urine 8-OHdG level was also adjusted by the urine creatinine level.

Peripheral endothelial function was assessed using an Endo-PAT 2000 device (Itamar Medical, Caesarea, Israel), as described previously^32^. Data were digitised and computed automatically using the Endo-PAT 2000 software; the RHI, representing the endothelial function, was defined as the ratio of the mean post-deflation signal (in the 90 to 120-second post-deflation interval) to the baseline signal in the hyperemic finger normalised by the same ratio in the contra-lateral finger and multiplied by a baseline correction factor, as calculated using the Endo-PAT 2000 software.

A standardised medical history was obtained, and an accurate physical examination was performed in all patients. Smoking status and medications were also recorded. Height and weight were measured in the standing position without shoes. BMI was calculated as the weight (kg) divided by the height (m) squared. After a 12-h fasting (no alcohol consumption), peripheral blood samples were collected. The creatinine, uric acid, urea nitrogen, fasting plasma glucose, glycosylated haemoglobin, total cholesterol (TC), triglycerides (TG), high-density lipoprotein cholesterol (HDL-C), LDL-C, and hs-CRP levels were measured using standard assays in all patients. The serum creatinine levels were assayed via a direct enzyme method (Biote Co., Ltd., Nanchang, China). The serum uric acid levels were also assayed via a direct enzyme method (Medical Co., Ltd., Ningbo, China). The serum urea nitrogen levels were assayed via the urease-GLDH method (Biote Co., Ltd., Nanchang, China). The fasting plasma glucose level was measured via the hexokinase/glucose-6-phosphate dehydrogenase method (Biote Co., Ltd., Nanchang, China). The glycosylated haemoglobin level was measured using high-performance liquid chromatography assay (Bio-Rad, USA). The TC levels were determined using enzymatic colorimetric assay (Medical Co., Ltd., Ningbo, China), TG levels using the GPO-POD method (Beckman Coulter, Suzhou, China), and HDL-C and LDL-C levels using the direct homogeneous assay methods with detergents (Medical Co., Ltd., Ningbo, China). The hs-CRP level was measured using the turbidimetric inhibition immunoassay (Beckman Coulter, Suzhou, China). All the biochemical variables were measured using an auto-analyser (OLYMPUS AU-2700) at the central laboratory of the Second Affiliated Hospital of Nanchang University.

The 24-h mean heart rate and 24-h SBP and DBP were assessed using 24-h dynamic electrocardiogram (Mini Holter Recorder, BI9800, Biomedical Instruments Co., Ltd, Shenzhen, China) and 24-h ambulatory blood pressure (BP) monitoring (Schiller Br-102 Plus Ambulatory BP Monitor, SCHILLER Americas Inc., USA), respectively.

### Statistical analysis

Normally distributed results were expressed as means ± standard deviations. The fasting plasma glucose, hs-CRP, and creatinine levels, right atrial diameter, right ventricular end-diastolic diameter, left ventricular end-diastolic posterior wall thickness, LVMI, and left ventricular ejection fraction were not normally distributed and were expressed as medians (interquartile ranges). Categorical values were presented as numbers (percentages).

Differences among groups were evaluated using one-way analysis of variance, followed by the post hoc test with Least-Significant Difference (LSD) for the continuous variables and the chi-square test for the categorical variables. The univariate and stepwise multivariate linear regression analyses were performed to assess the relationship between the clinical factors and eGFR. Continuous variables with a skewed distribution were natural logarithm (ln)-transformed to attain normal distributions. The Pearson’s correlation was used for simple linear analysis between the creatinine-adjusted urine 8-OHdG level and AHI and eGFR. A two-sided P value of < 0.05 was considered significant. All statistical analyses were performed using SPSS software for Windows, version 16.0 (SPSS, Chicago, IL, USA).

### Data Availability

All data generated or analysed during this study are included in this published article.
